# Evaluation of the correlation of MACC1, CD44, Twist1, and KiSS-1 in the metastasis and prognosis for colon carcinoma

**DOI:** 10.1186/s13000-018-0722-z

**Published:** 2018-07-18

**Authors:** Bo Zhu, Yichao Wang, Xiaolin Wang, Shiwu Wu, Lei Zhou, Xiaomeng Gong, Wenqing Song, Danna Wang

**Affiliations:** 1 Department of Pathology, The First Affiliated Hospital of Bengbu Medical College, Bengbu, China; 2 Department of Pathology, Bengbu Medical University, Bengbu, China

**Keywords:** CAC, MACC1, CD44, Twist1, KiSS-1

## Abstract

**Background:**

Metastasis-associated in colon cancer 1 (MACC1) has been reported to promote tumor cell invasion and metastasis. Cancer stem cells and epithelial-mesenchymal transition (EMT) have also been reported to promote tumor cell proliferation, invasion, and metastasis. KiSS-1, a known suppressor of metastasis, has been reported to be down-regulated in various tumors. However, the associations of MACC1, CD44, Twist1, and KiSS-1 in colonic adenocarcinoma (CAC) invasion and metastasis remain unclear. The purpose of this study is to investigate the roles of MACC1, CD44, Twist1, and KiSS-1 in CAC invasion and metastasis and their associations with each other and with the clinicopathological characteristics of CAC patients.

**Methods:**

Immunohistochemistry and multivariate analysis were carried out to explore the expression of MACC1, CD44, Twist1, and KiSS-1 in 212 whole-CAC-tissue specimens and the corresponding normal colon mucosa tissues. Demographic, clinicopathological, and follow-up data were also collected.

**Results:**

The results of this study showed MACC1, CD44, and Twist1 expression to be up-regulated, and KiSS-1 expression was down-regulated in CAC tissues. Positive expression of MACC1, CD44, and Twist1 was found to be positively correlated with invasion, tumor grades, and lymph- node-metastasis (LNM) stages and tumor-node-metastasis (TNM) stages for patients with CAC. Positive expression of KiSS-1 was inversely associated with invasion, tumor size, LNM stage, and TNM stage. The KiSS-1-positive expression group had significantly more favorable OS than did the KiSS-1-negative group. Univariate analysis indicated that overexpression of MACC1, CD44, and Twists1 was negatively associated with longer overall survival (OS) time, and there was a positive relationship between KiSS-1-positive expression and OS time for patients with CAC. Multivariate Cox analysis demonstrated that overexpression of MACC1, CD44, Twist1, and low expression of KiSS-1 and LNM and TNM stages were independent predictors of prognosis in patients with CAC.

**Conclusions:**

The results in this study indicated that levels of expression of MACC1, CD44, Twist1, and KiSS-1 are related to the duration of OS in patients with CAC. MACC1, CD44, Twist1, and KiSS-1 may be suitable for use as biomarkers and therapeutic targets in CAC.

## Background

Colorectal cancer (CRC) is the third most common cancer worldwide, with an estimated 1.4 million new cases in 2012 [1]. In China, CRC had an estimated 376,300 cases, which made it the fifth most common cancer in 2015 [2]. The most common causes of cancer treatment failure are relapse and metastasis. This may be related to an oncogene called metastasis-associated in colon cancer 1 (MACC1). In 2009, MACC1 was first found in colon cancer cell lines [3]. MACC1 is reported to combine with the mesenchymal-epithelial transition (MET) gene promoter and so participate in the hepatocyte growth factor/mesenchymal-epithelial transition (HGF/MET) signaling pathway [3, 4]. MACC1 is also reported to not only promote tumor cell proliferation, invasion, and dissemination by inducing epithelial-mesenchymal transition (EMT) in vitro [5, 6] but also to induce tumor cell growth, invasion, and metastasis in vivo [3, 7]. It has been demonstrated that MACC1 should be defined as a prognostic and metastatic biomarker for various cancers [8].

Many more studies have ascribed tumor metastasis and recurrence to a subpopulation of tumor cells defined as cancer stem cells (CSCs, also called tumor-initiating cells). CSCs have the characteristics of self-renewal, proliferation, invasiveness, and metastasis. They are responsible for cancer initiation and natural resistance to therapy [8–12]. CD44 is not only a common biomarker of CSCs in cancers, such as colorectal cancer, lung cancer, and glioblastoma [13–15], but also a receptor of hyaluronan. CD44 levels are correlated with cell-to-extracellular matrix (ECM) adhesion, cell growth, and angiogenesis [16, 17].

It has been demonstrated that cancer cells can invade and metastasize after they lose epithelial features and gain a mesenchymal phenotype, which is called the epithelial-mesenchymal transition (EMT) [18, 19]. Twist1, which belongs to the highly conservative basic helix-loop-helix family, is a transcription factor. Twist1 is a pivotal regulator of EMT and reported to promote N-cadherin synthesis and inhibit E-cadherin expression [20, 21], thus causing profound morphological changes in tumor cells and expression of cell-matrix adhesion genes to induce tumor cell mobility and migration [22].

KiSS-1, which was originally identified in non-metastatic melanoma by analysis of subtractive hybridization, is widely considered a critical cancer metastasis suppressor gene [11, 23]. The KiSS-1 gene, which encodes a 145-amino-acid protein, can bind to the G protein-coupled receptor 54 (GPR54, also called KiSS-1R). KiSS-1 can control cell-cell adhesion by promoting E-cadherin expression and cell-matrix adhesion and cytoskeleton remodeling through inhibition of MMP expression [11, 24, 25]. KiSS-1 expression is also reported to suppress the metastatic potential of tumor cells but not tumorigenicity [25, 26]. Further studies have demonstrated that downregulation of KiSS-1 may be involved in the process of tumor invasiveness and metastasis [11, 25, 26].

The purpose of the current study is to assess the expression of MACC1, CD44, Twist1, and KiSS-1 in the colonic adenocarcinoma (CAC) tissues of patients and their associations between pathological characteristics and prognosis of patients with CAC. Immunohistochemistry was used to evaluate the expression of MACC1, CD44, Twist1, and KiSS-1 in CAC tissues and the corresponding adjacent normal colon mucosa tissues of patients with CAC.

## Methods

### Patients and tissue specimens

We collected the records of 212 patients (median age: 56.6 years; and range: 29–78 years) with CAC (rectal adenocarcinomas were excluded) diagnosed at the Department of Pathology at our hospital from January 2010 to December 2011. Because all outcomes had already taken place before the study began, it is retrospective. Patients who had any history of anti-cancer therapy were excluded. All patients with CAC provided written, extensively informed consent for their specimens to be used (including in hospital and out hospital). The study was carried out in accordance with the Declaration of Helsinki guidelines and approved by the Bengbu Medical College ethics committee (No. BBMCEC2016024). We collected patient data including complete clinicopathological, demographic, and follow-up data (follow-up at 3-month intervals through mobile phone or social applications). Overall survival (OS) time was computed from the date of radical surgery to date of death or to December 2016 (their mean OS: 53.3 months; and range: 22–72 months). TNM stages and LNM stages were calculated in accordance with the 8th edition of the guidelines issued by the American Joint Committee on Cancer (AJCC). Tumor grades were calculated in accordance with the standards issued by the World Health Organization (WHO). Specific clinicopathological characteristics are shown in Table 1.

**Table 1:** Patients characteristics

Patients characteristics	Frequency (*n*)	Percentage (%)
Gender		
Male	142	67.0
Female	70	33.0
Ages		
≤ 60	134	63.2
> 60	78	36.8
Size		
≤ 2.0 cm	33	15.6
> 2.0 cm, ≤5.0 cm	110	51.9
> 5.0 cm	69	32.5
Location		
Ascending	42	19.8
Transverse	64	30.2
Descending	33	15.6
Sigmoid	73	34.4
Gross type		
Ulcerative	65	30.7
Infiltrating	46	21.7
Polypoid	68	32.1
Colloid	33	15.6
Invasion		
Submucosa	36	17.0
Muscularis	64	30.2
Subserosa^a^	101	47.6
Visceral peritoneum^b^	11	5.2
Grade		
Well	32	15.1
Moderate	135	63.7
Poor	45	21.2
Lymph node metastasis stages		
N0	136	64.2
N1	70	33.0
N2	6	2.8
TNM stage		
I	69	32.5
II	67	31.6
III	76	35.8

^a^The tumor has grown through the muscularis propria and into the subserosa, which is thin layer of connective tissue beneath the outer layer of some parts of the large intestine, or it has grown into tissues surrounding the colon. ^b^ The tumor has grown into the surface of the visceral peritoneum, which means it has grown through all layers of the colon, or the tumor has grown into or has attached to other organs or structures

### Immunohistochemistry

All tissues were fixed in 10% buffered formalin solution and then embedded in paraffin. All tissues were then cut into 4-μm-thick sections. Immunostaining was conducted using the Elivision™ Plus method, and the procedure was performed in accordance with the kit instructions. Samples were deparaffinized using routine methods and dehydrated using xylene and alcohol. Methanol containing 3% H_2_O_2_ solution was used for blocking endogenous peroxidase activity, and citrate buffer was used to repair antigen. Goat serum was used for blocking. MACC1 (rabbit polyclonal antibody, Santa Cruz Biotechnology, US), CD44 (mouse monoclonal antibody, Abcam, US), Twist1 (mouse monoclonal antibody, Abcam, US), and KiSS-1 (mouse monoclonal antibody, Santa Cruz Biotechnology, US) primary antibodies were added, and then all sections were incubated overnight at 4 °C. Then enhancer (reagent A) and reagent B were added. The images were allowed to develop in diaminobenzidine (DAB) substrate. Finally, all sections were re-dyed with hematoxylin and mounted with gum.

### Assessment of immunostaining

Ten randomly selected high-power-field (HPF) fields of every CAC section were selected to forestall any intratumoral heterogeneity of marker expression. In accordance with percentage of positive cells and positive intensity, immunostaining results were multiplied using intensity scores (0 points means none; 1 point means weak staining; 2 points means moderate staining; 3 point means strong staining) and percentage scores (1 point is positive cells ≤10%; 2 points is 10% < positive cells ≤50%; 3 points is 50% < positive cells ≤75%; 4 points is positive cells > 75%) which ranged from 0 to 12 [8, 11]. Here > 2 points was considered indicative of positive expression. For slices positive for all of biomarkers, the average score of all sections was taken.

### Statistical analysis

All data were analyzed using SPSS 19.0 software (Chicago, IL, US). Countable data were subjected to the Chi-square test for comparisons between two groups. Multivariate logistic regression analysis was performed to establish the relative factors for metastasis. Univariate OS analysis was carried out using the Kaplan-Meier method with log-rank test. Multivariate OS analysis was carried out using Cox regression model test. *P* < 0.05 was considered indicative of statistically significant differences.

## Results

### Associations between MACC1, CD44, Twist1, and KiSS-1 in cancer tissues of patients and clinicopathological characteristics

**Fig. 1:**
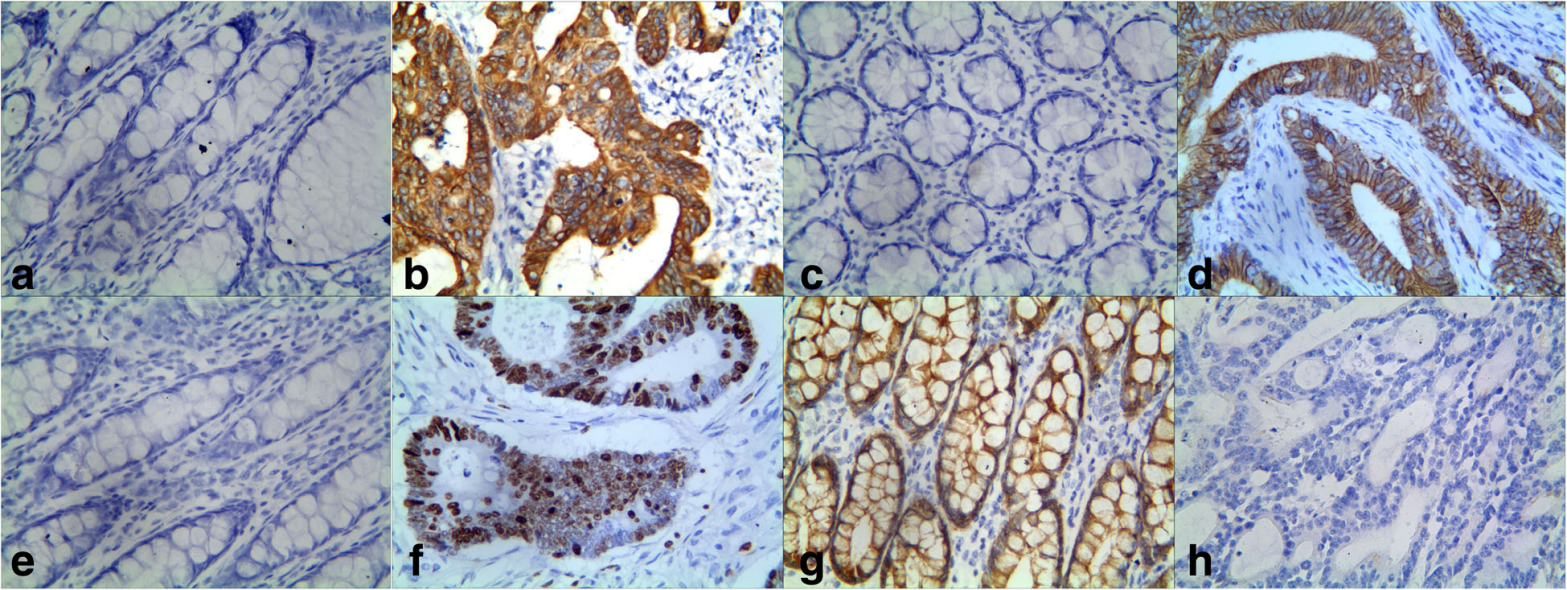
Immunostaining for MACC1, CD44, Twist1, and KiSS-1 in colon adenocarcinoma and control tissue. **a**: Negative MACC1 in the control tissue (400 magnification); **b**: Positive MACC1 in the CAC tissue (400 magnification); **c**:Negative CD44 in the control tissues (400 magnification); **d**: Positive CD44 in the membrane and cytoplasm of cancer cells (400 magnification); **e**: Negative Twist1 in the control tissue (400 magnification); **f**: Positive Twist1 in the cytoplasm and nuclei of the cancer cells (100 magnification); **g**: Positive KiSS-1 in the cytoplasm of control cells (400 magnification); **h**: Negative KiSS-1 in the cancer tissue (400 magnification)

As shown in Fig. 1a, b, MACC1-positive expression was mainly confined to the cytoplasm. The positive expression of MACC1 in the CAC specimens (61.3%, 130/212) was significantly higher than in the normal colon mucosa specimens (7.1%, 15/212; *P* < 0.001). The immunostaining results indicated that positive expression of MACC1 in CAC was positively correlated with invasion, tumor differentiation, LNM stages, and TNM stages (Table 2).

**Table 2:** The correlation between MACC1, or CD44, or Twist1, or KiSS-1 and clinicopathological characteristics in colon adenocarcinoma

Variable	MACC1		*P*	CD44		P	Twist1		P	KiSS-1		*P*
	Negative	Positive		Negative	Positive		Negative Positive			Negative	Positive	
Gender			0.239			0.929			0.783			0.564
Male	51	91		64	78		50	92		87	55	
Female	31	39		32	38		26	44		40	30	
Age (years)			0.263			0.927			0.775			0.833
≤60	48	86		61	73		49	85		81	53	
> 60	34	44		35	43		27	51		46	32	
Size (cm)			0.081			0.375			0.085			< 0.001
≤2.0	10	23		14	19		10	23		16	17	
> 2.0, ≤5.0	38	72		46	64		34	76		80	30	
> 5.0	34	35		36	33		32	37		31	38	
Location			0.863			0.156			0.507			0.526
Ascending	17	25		21	21		13	29		21	21	
Transverse	22	42		25	39		22	42		39	25	
Descending	13	20		11	22		10	23		21	12	
Sigmoid	30	43		39	34		31	42		46	27	
Gross type			0.056			0.406			0.002			0.929
Ulcerative	27	38		35	30		31	34		37	28	
Infiltrating	10	36		20	26		6	40		28	17	
Polypoid	29	39		28	40		25	43		41	27	
Colloid	16	17		13	20		14	19		21	12	
Invasion			0.035			0.040			0.002			0.003
Submucosa	20	16		21	15		19	17		13	23	
Muscularis	19	45		29	35		30	34		38	26	
Subserosa	41	60		45	56		25	76		66	35	
Visceral peritoneum	2	9		1	10		2	9		10	1	
Grade			0.032			0.024			< 0.001			0.745
Well	18	14		20	12		19	13		18	14	
Moderate	52	83		62	73		53	82		80	55	
Poor	12	33		14	31		4	41		29	16	
LNM stages			< 0.001			< 0.001			< 0.001			< 0.001
N0	71	65		85	51		64	72		60	76	
N1	11	59		11	59		12	58		61	9	
N2	0	6		0	6		0	6		6	0	
TNM stage			< 0.001			< 0.001			< 0.001			< 0.001
I	37	32		47	22		40	29		23	46	
II	34	33		38	29		24	43		37	30	
III	11	65		11	65		12	64		67	9	

As shown in Fig. 1c, d, CD44 positive expression was mainly confined to the cell membrane and cytoplasm. Similar to MACC1, the positive expression of CD44-positive expression in CAC tissues (54.7%, 116/212) was significantly greater than in the normal colon mucosa tissues (16.5%, 35/212; *P* < 0.001). The results also demonstrated that positive expression of CD44 in CAC was positively correlated with invasion, tumor differentiation, LNM stages, and TNM stages (Table 2).

As shown in Fig. 1e, f, Twist1 expression was mainly confined to the cytoplasm and nuclei. The expression of Twist1 in CAC tissues (64.2%, 136/212) was significantly greater than in the normal colon mucosa tissues (9.4%, 20/212; *P* < 0.001). The results also showed that Twist1 expression in CAC was significantly closely associated with tumor differentiation, gross type, invasion, LNM stages, and TNM stages (Table 2).

As shown in Fig. 1g, h, KiSS-1-positive expression was mainly confined to the cytoplasm. The positive expression of KiSS-1 in CAC tissues (40.1%, 82/212) was significantly lower than in the normal colon mucosa tissues (94.3%, 200/212; *P* < 0.001). The results indicated that positive expression of KiSS-1 was inversely correlated with tumor size, invasion, LNM stages, and TNM stages (Table 2).

### Associations among MACC1, CD44, Twist1, and KiSS-1 in CAC

The association between KiSS-1 expression and MACC1, CD44, and Twist1 expression was found to be negative (*r* = − 0.437; *r* = − 0.397; *r* = − 0.251; respectively; *P* < 0.001) (Table 3). The association between MACC1 expression and CD44 expression and Twist1 expression was found to be positive (*r* = 0.270, *P* < 0.001; *r* = 0.315, *P* < 0.001). The association between CD44 expression and Twist1 expression was found to be positive (*r* = 0.150, *P* = 0.029) (Table 3).

**Table 3:** Correlation among MACC1, CD44, Twist1 and KiSS-1 in CAC

Variable	MACC1		r	*P*	CD44		r	*P*	KiSS-1		r	*P*
	Negative	Positive			Negative	Positive			Negative	Positive		
MACC1							0.270	< 0.001^a^			−0.437	< 0.001^b^
Negative					51	31			27	55		
Positive					45	85			100	30		
CD44			0.270	< 0.001^a^							−0.397	< 0.001^b^
Negative	51	45							37	59		
Positive	31	85							90	26		
Twist1			0.315	< 0.001^a^			0.150	0.029^a^			−0.251	< 0.001^b^
Negative	45	31			42	34			33	43		
Positive	37	99			54	82			94	42		

^a^positive correlation, ^b^negative correlation

### Metastasis

Univariate metastasis analysis indicated that invasion was positively correlated with LNM stages (*P* < 0.05). Multivariate metastasis logistic analysis suggested that overexpression of MACC1, CD44, Twist1, and down-regulation of KiSS-1 and invasion were both significantly closely associated with LNM (Table 4).

**Table 4:** Univariate analysis and multivariate analysis of factors affecting lymph node metastasis

Variables	Categories	Univariate analysis	Multivariate analysis		
		*P*	HR	95% CI	*P*
Invasion	Subserosa/ Visceral peritoneum^a^	< 0.001	12.336	1.264-120.427	0.031
MACC1	Negative/Positive	< 0.001	2.956	1.222–7.149	0.016
CD44	Negative/Positive	< 0.001	6.496	2.858–14.767	< 0.001
Twist1	Negative/Positive	< 0.001	3.951	1.673–9.331	0.002
KiSS-1	Negative/Positive	< 0.001	0.271	0.110–0.666	0.004

^a^Subserosa: The tumor has grown through the mucosa and into the subserosa; Visceral peritoneum: The tumor has grown into the surface of the visceral peritoneum, which means it has grown through all layers of the colon, or the tumor has grown into or has attached to other organs or structures

### Survival analysis

**Fig. 2:**
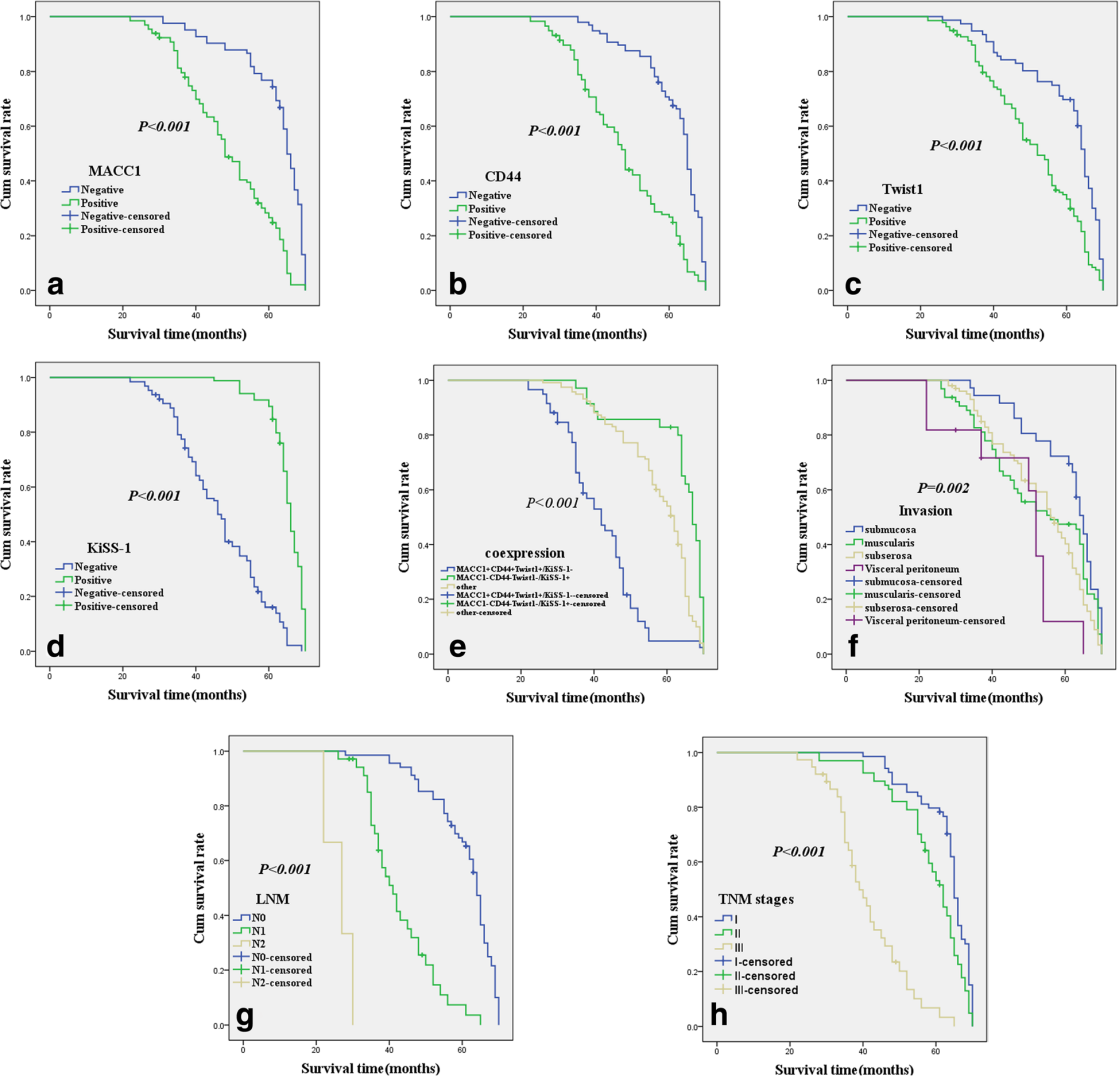
Kaplan-Meier analysis curve of the survival rate of patients with CAC. The y-axis means the percentage of patients; the x-axis means their survival in months. **a**: OS analysis of all patients in relation to MACC1 (log-rank = 61.757, *P* < 0.001); **b**: OS analysis of all patients in relation to CD44 expression (log-rank = 54.938, *P* < 0.001); **c**: OS analysis of all patients in relation to Twist1 expression (log-rank = 24.306, *P* < 0.001); **d**: OS analysis of all patients in relation to KiSS-1 expression (log-rank = 115.258, *P* < 0.001). In **(a)**, (**b)**, (**c)** and (**d)** analyses, the green line represents patients with positive MACC1, or CD44, or Twist1 or KiSS-1; the blue line representing the negative MACC1, or CD44, or Twist1 or KiSS-1 group. E: OS survival of all patients in relation to the combination of KiSS-1, MACC1, CD44, and Twist1 expression (log-rank = 84.625, *P* < 0.001). The green line represents negative KiSS-1 and positive MACC1, CD44, Twist1 and the blue line represents positive KiSS-1 and negative MACC1, CD44, Twist1. The brown line represents other positive or negative proteins. F: OS survival of all patients in relation to tumor invasion depth (log-rank = 14.868, *P* = 0.002); G: OS survival of all patients in relation to LNM stages (log-rank = 325.068, *P* < 0.001); H: OS survival of all patients in relation to TNM stages (log-rank = 152.179, *P* < 0.001)

As shown in Fig. 2a, univariate OS analysis indicated that the OS time of MACC1+ (47.8 ± 12.5 months) for patients with CAC was significantly shorter than that of MACC1- for patients (62.0 ± 9.6 months; log-rank = 61.757, *P* < 0.001). As shown in Fig. 2b, the univariate OS time of CD44+ (46.8 ± 12.9 months) was significantly lower than in CD44- patients (61.2 ± 8.9 months; log-rank = 54.938, *P* < 0.001). As shown in Fig. 2c, the univariate OS time of Twist1+ patients (49.7 ± 13.0 months) was significantly lower than in Twist1- patients (59.7 ± 11.5 months; log-rank = 24.306, *P* < 0.001). As shown in Fig. 2d, the univariate OS time of KiSS-1+ patients (64.7 ± 4.9 months) was significantly greater than that of KiSS-1- patients (45.7 ± 11.6 months; log-rank = 115.258, *P* < 0.001). As shown in Fig. 2e, the univariate OS time of the combination of KiSS-1 negative expression and MACC1+, CD44+, and Twist1+ positive expression patients was significantly lower than that in KiSS-1 positive expression and MACC1-, CD44-, and Twist1- (log-rank = 84.625, *P* < 0.001). The univariate OS time was also significantly closely associated with the following other clinicopathological characteristics, invasion (*P* = 0.002, log-rank = 14.868; Fig. 2f), LNM stages (*P* < 0.001, log-rank = 325.068; Fig. 2g), and TNM stages (*P* < 0.001, log-rank = 152.179; Fig. 2h) (Table 5).

**Table 5:** Results of univariate analyses of overall survival (OS) time

Variable	n	Mean OS (months)	Log-rank	*P* value
MACC1			61.757	< 0.001
Negative	82	62.0 ± 9.6		
Positive	130	47.8 ± 12.5		
CD44			54.938	< 0.001
Negative	96	61.2 ± 8.9		
Positive	116	46.8 ± 12.9		
Twist1			24.306	< 0.001
Negative	76	59.7 ± 11.5		
Positive	136	49.7 ± 13.0		
KiSS-1			115.258	< 0.001
Negative	127	45.7 ± 11.6		
Positive	85	64.7 ± 4.9		
Gender			0.070	0.792
Male	142	54.0 ± 12.4		
Female	70	52.0 ± 15.2		
Ages (year)			0.206	0.650
≤ 60	134	54.3 ± 12.7		
> 60	78	51.7 ± 14.4		
Size (cm)			5.887	0.053
≤ 2.0	33	55.0 ± 13.4		
> 2.0, ≤5.0	110	51.0 ± 12.7		
> 5.0	69	56.2 ± 13.9		
Location			7.503	0.057
Ascending	42	54.3 ± 14.0		
Transverse	64	51.4 ± 13.4		
Descending	33	52.3 ± 13.3		
Sigmoid	73	54.9 ± 13.0		
Type			3.781	0.286
Ulcerative	65	55.3 ± 13.0		
Infiltrating	46	51.8 ± 13.4		
Polypoid	68	52.6 ± 14.0		
Colloid	33	53.2 ± 12.7		
Invasion			14.868	0.002
Submucosa	36	60.2 ± 9.9		
Muscularis	64	52.1 ± 14.7		
Subserosa	101	52.7 ± 12.5		
Visceral peritoneum	11	43.2 ± 14.4		
Grade			3.544	0.170
Well	32	56.1 ± 14.1		
Moderate	135	53.7 ± 13.0		
Poor	45	50.1 ± 13.5		
LNM stages			325.068	< 0.001
N0	136	60.6 ± 8.8		
N1	70	41.5 ± 9.1		
N2	6	26.3 ± 3.6		
TNM stage			152.179	< 0.001
I	69	62.7 ± 7.4		
II	67	58.4 ± 9.7		
III	79	40.3 ± 9.7		

Multivariate analysis suggested that MACC1, CD44, Twist1, and KiSS-1 expression, LNM stages, and TNM stages should be considered independent predictors affecting patient survival (Table 6).

**Table 6:** Results of multivariate analyses of overall survival (OS) time

Variable	B	SE	*P*	RR	95% CI
MACC1	1.070	0.198	< 0.001	2.917	1.978–4.301
CD44	0.512	0.176	0.004	1.669	1.183–2.357
Twist1	0.348	0.176	0.048	1.417	1.003–2.000
KiSS-1	−1.368	0.201	< 0.001	0.255	0.172–0.377
LNM stages	0.801	0.368	0.029	2.229	1.084–4.581
TNM stages	0.630	0.260	0.015	1.877	1.127–3.124

## Discussion

Colon cancer is a common malignant tumor of the digestive system. Its high heterogeneity makes it difficult to fully evaluate the comprehensiveness and effectiveness of any biomarker. Previous studies have demonstrated that MACC1 can promote tumor cell proliferation and migration [3, 4]. In this study, our findings indicated that positive expression of MACC1 in CAC was positively correlated with invasion and tumor differentiation and LNM and TNM stages. Positive expression of MACC1 was found to be significantly closely associated with lower OS time when compared with MACC1 negative. These findings demonstrated that MACC1 was considered an effective biomarker for invasion and metastasis, as well as a predictor for prognosis [3–8, 27, 28].

CD44 was initially considered an adhesion molecule capable of regulating cell-ECM adhesion, invasion, and metastasis [16, 17]. CD44 overexpression has been found to be correlated with tumorigenesis and to predict a poor response to anti-cancer therapy [8, 15]. The results recorded in this study also demonstrated that positive expression of CD44 in CAC was positively correlated with tumor differentiation, invasion, LNM stages, and TNM stages. CD44+ patients showed shorter OS times than CD44- patients. Several other studies have explored the metastatic and prognostic significance of CD44, and they produced similar results [16, 17]. These findings confirmed that CD44 may be an effective biomarker for predicting the invasion and metastasis of CAC and may predict prognosis.

EMT is believed to be involved in a series of fundamental biological behaviors, such as growth, motility, invasion, adhesion, metastasis, and recurrence. Twist1, which consists of two exons and one intron, is a pivotal transcriptional factor in EMT. The results of this study showed the expression of Twist1 in CAC to be positively associated with tumor differentiation, gross type, invasion, and LNM stages and TNM stages. Twist1+ patients showed significantly shorter OS than Twist1- patients. Because the infiltrating type of CAC tends to develop more rapidly than other types of CAC, which could suggest that Twist1 is a valuable biomarker for more aggressive CAC. In this way, our findings support the conclusion that Twist1 may be a reliable biomarker of CAC, particularly in predicting progression, metastasis, and prognosis.

KiSS-1 is considered a metastatic suppressor in many cancers [11, 25, 26]. Results have demonstrated that the normal expression of KiSS-1 can suppress tumor cell growth, motility, and migration. The results of this study indicated a negative correlation between positive expression of KiSS-1 and tumor size, invasion, LNM stage, or TNM stage. KiSS-1+ patients were significantly associated with longer OS time when compared with KiSS-1- patients. These findings suggested that KiSS-1 should be considered as a potential predictor for progression and metastasis of CAC, as well as prognosis [11, 25, 26, 29].

In the current study, univariate analysis indicated that invasion, LNM, TNM stages, and expression of MACC1, CD44, Twist1, and KiSS-1 were significantly closely associated with duration of OS in patients with CAC. Multivariate OS analysis showed that LNM stages, TNM stages, positive expression of MACC1, CD44, Twist1, and KiSS-1 were independent predictors affecting patient survival. Multivariate metastasis logistic analysis showed expression of MACC1, CD44, Twist1, and KiSS-1, and invasion to be significantly closely associated with metastasis of CAC. These findings also demonstrated that MACC1, CD44, Twist1, and KiSS-1 should be considered to be useful biomarkers for predicting the invasion and metastasis of CAC, as well as a predictor for prognosis.

There were some differences between our results and previous findings. This may be related to the use of different biomarkers, different immunohistochemical methods, or even different patients (such as Wang W. et al., who reported that KiSS-1 expression was statistically significantly higher in colorectal cancer tissue than in corresponding adjacent normal mucosa [30], Wu Q. et al. reported that CD44 expression was not associated with LNM in multivariate logistic regression analysis [17], and Yusup A. et al. reported that Twist1 expression was not correlated with survival [31]). However, we demonstrated that MACC1, CD44, Twist1, and KiSS-1 expression were associated with metastasis and prognosis of CAC. CSCs may indicate the initiation, progression, and metastasis of CAC. Their capacity for self-renewal, proliferation, and multiple forms of differentiation allow CSCs to induce EMT and so promote invasion and metastasis. CD44 is an adhesion molecule that can regulate cell-matrix adhesion. CD44 overexpression is beneficial to CAC progression and metastasis. During cancer progression, MACC1 overexpression should inhibit cancer cell apoptosis and promote cancer cell EMT via HGF/MET pathways [32, 33]. Twist1 overexpression could further promote the cancer cell EMT process through regulating E-cadherin, N-cadherin, and MMP expression [21, 34]. Thus, EMT could induce cancer cell motility, migration, and even metastasis. Aberrant expression of KiSS-1 could decrease or lower its ability to inhibit cancer cell invasion and metastasis [11, 25, 26].

## Conclusions

This study demonstrated that expression levels of MACC1, CD44, Twist1, and KiSS-1 are related to duration of OS among patients with CAC. In this way, MACC1, CD44, Twist1, and KiSS-1 could serve as valuable biomarkers in CAC and may be helpful for the metastasis and prognosis for CAC.
